# Assessment of Brainstem Functional Organization in Healthy Adults and Overactive Bladder Patients Using Ultra-High Field fMRI

**DOI:** 10.3390/biomedicines11020403

**Published:** 2023-01-29

**Authors:** Susana Fernández Chadily, Mathijs M. de Rijk, Janine M. W. Janssen, Job van den Hurk, Gommert A. van Koeveringe

**Affiliations:** 1 Department of Urology, School for Mental Health and Neuroscience, Faculty of Health, Medicine and Life Sciences, Maastricht University, 6229 ER Maastricht, The Netherlands; 2 Department of Urology, Maastricht University Medical Center+ (MUMC+), 6229 HX Maastricht, The Netherlands; 3 Scannexus Ultra High-Field MRI Center, 6229 EV Maastricht, The Netherlands

**Keywords:** overactive bladder syndrome, neuro-urology, fMRI, PAG

## Abstract

The pathophysiological mechanisms of overactive bladder syndrome (OAB) remain largely unknown, with major involvement of the central nervous system (CNS). The periaqueductal gray (PAG) is a brainstem area which is indicated to play an essential role in bidirectional communication between the bladder and the CNS. We aimed to assess consistency of PAG functional organization across different bladder sensory states in OAB patients. We propose, that PAG functional organization patterns across sensory states will differ between controls and OAB patients. We analyzed fMRI scans at 7 Tesla from six controls and two OAB patients. The Louvain module detection algorithm was applied to parcellate the PAG in empty and full bladder states. We assessed within-subject consistency and investigated differences in this consistency between both groups. High within-subject agreement of PAG parcellations between empty and full bladder states was demonstrated in both groups. Additionally, we showed that the correlations between PAG clusters in both bladder states were significantly different in patients compared to controls (*p* = 0.039). The methods introduced here offer a promising tool to assess functional organization of the PAG and understand the underlying pathology and the role of this region in OAB syndrome.

## 1. Introduction

Overactive bladder (OAB) syndrome is characterized by urinary urgency, frequency, and nocturia, with or without urinary incontinence that is not caused by a urinary tract infection or other obvious pathology [1]. It can have a detrimental impact on the quality of life and affect social, sexual, occupational, and psychological aspects of a patient’s life [2]. In a large population-based survey in five European countries, Irwin et al. estimated the prevalence of OAB to be 11.8% with similar rates in men and women [2]. Of all types of urinary incontinence, OAB was more prevalent than all other types combined [2]. A study from 2006 estimated that the average annual direct costs per patient per year of OAB management (medical visits, incontinence pads, drugs, UTIs, skin conditions, and falls and fractures) ranged from €269 to €706, with an average of 63% of the costs accounting for incontinence pads [3]. By 2020, the total costs of the healthcare system across Canada, Germany, Italy, Sweden, and the United Kingdom were estimated to be €5.2 billion [3].

A wide range of therapeutic approaches is available with success rates up to 70%. These approaches include lifestyle changes, pelvic floor physiotherapy, drug therapy, bladder botulinum toxin injections, and sacral neuromodulation. While the majority of patients benefit from conservative measures, those patients with refractory symptoms will require more invasive options.

The neural network involved in the control of bladder activity is suggested to be affected in OAB patients [4]. For instance, Clarkson et al. suggest that there may be at least two mechanisms that contribute to urinary incontinence. One in which a breakdown in central control is the prime problem and is more responsive to behavioral therapy, and another in which central control is mainly intact and brain behavior and brain approaches may therefore be less useful as therapeutic targets [4].

In a healthy state, the storage and elimination of urine depend on coordinated activity between the bladder wall, the bladder neck, the urethra, and the urethral sphincter. These functional units form the lower urinary tract (LUT). Not only is the brain is involved in the integration of information, but the spinal cord and peripheral ganglia as well. LUT neural circuits are organized in a hierarchical system in which many circuits exhibit switch-like patterns in an all-or-none process [5]. A key structure within the brain–bladder pathways is the periaqueductal gray (PAG), and several studies have shown the importance of this region in LUT control [5,6,7]. The PAG is located in the brainstem and is one of the evolutionarily most preserved regions of the brain [6]. This region serves as a bridge between higher decision-making centers and lower centers involved in reflexive micturition [6]. It receives afferent activity from the bladder, has projections to different parts of the forebrain, and sends efferent signals to the bladder and urethra via the pontine micturition center. Therefore, the PAG occupies a pivotal role in the bidirectional communication between the brain and bladder. Functional imaging studies have shown activation of this structure during the bladder filling and voiding process, highlighting the critical role the PAG plays in both processes [8,9]. It is suggested that the PAG may be involved in voiding dysfunction or incontinence in a wide spectrum of disorders, including stroke, multiple sclerosis, migraine, and Wernicke’s encephalopathy [6]. This association emphasizes the need to understand the structural and functional properties of the PAG in conditions where voiding and storage are impaired. 

In order to elucidate the interactions between brain and bladder activity, recent research has focused on the combination of reported bladder sensations and functional imaging. Specifically, functional connectivity studies of the brain (defined as the correlation of neural activity within and between different brain areas [10,11]) have highlighted how brain areas interact during a specific task, and unraveled how these interactions differ between disease states and changes with therapy [4]. As bladder control depends on a complex and extensive network of brain regions, dysfunction in different parts of the brain can lead to urinary complaints. This suggests that there could be several phenotypes of OAB patients requiring different treatments [12]. Since the PAG functions as a relay station, it might be that PAG activity will partially reflect abnormal activity in higher brain areas. Accordingly, if higher structures are impaired this could be observed in the PAG as well. 

Previous work by our group aimed to parcellate the PAG in healthy adults at 7-Tesla (T) resting-state fMRI (rs-fMRI) and used bladder sensations to assess functional connectivity changes between PAG clusters [13,14]. Brain parcellation is defined as the division of brain structures into a number of clusters, or parcels, that have high within-cluster similarity of the blood-oxygen-level-dependent (BOLD) signal. Resulting clusters are spatial domains with non-overlapping regions that share common characteristics, such as resting-state BOLD fluctuations [15,16]. Creating parcellations of specific regions within the brain is fundamental to understand not only structural brain organization but also function [15]. Resting-state-based parcellations are proposed to reflect an underlying anatomical construct [17]. This approach can be used to study connectivity changes between different subregions of the PAG related to changes in bladder activity and bladder sensations. The method can reliably parcellate the PAG and will enable to the investigation of possible changes in the functional connectivity of this region in pathological conditions [13]. 

In spite of previous efforts to unravel pathophysiological mechanisms underlying OAB, much remains unknown, especially the role of the PAG in OAB syndrome [18]. More importantly, it remains unknown which specific treatment is better for each patient. The path to effectively manage incontinence relies currently on a trial and error procedure, going through the different therapeutic options until an effective therapy is found. This tedious process increases the burden on patients and caregivers to find an adequate treatment for their complaints. In addition, it may expose the patient to more side effects of therapies than needed. 

In the current project, we explored a methodological approach to investigate how functional connectivity patterns of the PAG change with bladder sensations in OAB patients compared to healthy adults. We investigated changes in ultra-high field (7-T) fMRI-based parcellations of the PAG between empty and full bladder states and compared these changes between healthy adults and OAB patients. We hypothesized that PAG consistency of resting state functional connectivity related to bladder sensations in OAB patients differs compared to healthy adults.

## 2. Materials and Methods

The study is conducted according to the principles of the Declaration of Helsinki, in line with the Medical Research Involving Human Subjects Act (WMO), and was approved by the local Medical research ethics committee (METC). In total, we recruited a cohort of 12 healthy female participants, without any clinically significant history of LUT dysfunction, or neurological disease or dysfunction, judged by the medical investigator and six OAB patients. However, for further analyses, only six healthy subjects (S1–S6) and two OAB patients (P1, P2) were included (mean ± SD age, 41 ± 16 years; range 18–66 years). We chose to include only female participants in this project to control for gender as a confounding factor. The data of 10 participants had to be excluded due to limitations in the dataset for the proposed analyses.

Participants enrolled in the study completed a total of two visits; a screening visit followed by a scanning session. During the screening, participants signed the informed consent form before any study-related procedure took place. Participants were asked to fill out a 3-day micturition diary to obtain baseline values of voiding and drinking patterns and to familiarize participants with scoring their perception of urgency on the four-point Indevus Urgency Severity Scale (IUSS) [19]. 

During the scanning visit, participants visited the outpatient urology clinic (Maastricht University Medical Center, Maastricht, The Netherlands) where a transurethral filling catheter was placed after participants voided until empty in private. Additionally, urodynamic recordings were made during the fMRI session using the Laborie/MMS Luna ambulatory urodynamic device in order to monitor bladder activity during the scanning session. Upon arrival at the Ultra-High-Field MRI center (Scannexus, Maastricht, The Netherlands), participants were requested to lie down in a supine position on the MRI bed. Scans were acquired on a 7-T MRI scanner (Siemens, MAGNETOM, Erlangen, Germany) using a 32-channel head TxRx coil (NOVA Medical, Wilmington, MA, USA). An MRI-compatible syringe pump was connected to the filling catheter and foam cushioning was placed to restrict head motion. The region of interest (ROI) was the supramedullary portion of the brainstem, which included the PAG. 

Prior to starting with the scanning protocol, any residual urine was emptied via the catheter. In the current study, the use of rs-fMRI enables us to investigate spontaneous, slow-changing fluctuations of brain activity using a paradigm that to some extent resembles natural bladder filling. First, we conducted an empty bladder rs-fMRI scan with 420 T2*-weighted multiband echo planar imaging volumes (mb-EPI sequence, acceleration factor = 2, MB-factor = 2, TR = 1400 ms, TE = 22 ms, resolution = 1.1 × 1.1 × 1.1 mm), and 40 slices covering the ROI. Subsequently, a T1-weighted whole-brain anatomical scan using a magnetization prepared rapid gradient echo sequence (resolution 0.7 × 0.7 × 0.7 mm) was obtained. Then, the bladder was filled with a saline solution at body temperature with an automatic syringe pump at a speed of 30 mL/min. Participants reported their bladder fullness on a visual analog scale (VAS) and their urgency (IUSS) with an MRI-safe joystick. The reported bladder sensations were presented to the researchers in real time. When the reported urgency level was equal to 2, the bladder was no longer filled and a resting-state full bladder (strong desire to void) scan started. We collected 420 T2*-weighted multiband echo planar volumes (md-EPI sequence, acceleration factor = 2, MB-factor = 2, TR = 1400 ms, TE = 22 ms, resolution = 1.1 × 1.1 × 1.1 mm) and 40 slices of the ROI. Once the protocol finished, participants were assisted safely out of the scanner and instructed to void in private. The voided volume was recorded by the participants and reported to the researcher.

Imaging data were pre-processed using BrainVoyager 22.2 (Brain Innovation, Maastricht, The Netherlands). We created two functional documents for each participant, corresponding to the empty and full bladder scans. Pre-processing steps included a slice scan time correction, motion detection and correction, temporal high pass filtering, and correcting for geometric distortions due to non-zero off-resonance fields. 

We then created the anatomical volume data, extracted the brain tissue from the head scan, and performed intensity inhomogeneities corrections to improve homogeneity. Functional data were subsequently co-registered with the anatomical data, and we normalized the anatomical and functional data to MNI space, a standard spatial coordinate system. 

First, we manually segmented PAG in the MNI template brain and used this mask as a reference region of interest for subsequent analyses. These post-processing steps were performed using custom MATLAB (MathWorks, Natick, MA, USA) scripts.

Then, we computed connectivity matrices in both the empty and full bladder rs-fMRI scans, where the time course of each voxel was correlated in a pair-wise fashion. The lowest 5% correlations were removed with the aim to improve the data sets since we assumed they may represent weak and non-significant connections [20]. The Louvain module detection algorithm was used to generate clusters based on the connectivity profile of PAG, resulting in up to four clusters. These clusters represent a group of voxels within the PAG that has a highly similar fluctuation in the BOLD signal during the empty or full bladder resting state scan and is proposed to represent functional subdivisions of the PAG. Since the approach has a stochastic nature, meaning that the outcome involves certain variability, the algorithm was run for 500 iterations, and the parcellation with the largest Q-value (modularity statistic) was selected for further analysis [13]. Once the clustering was performed, we created PAG module maps for each bladder state and participant and assessed how similar each participant’s full bladder parcellation was to their empty bladder parcellation. We computed the (spatial) correlation coefficient between each participant’s empty and full bladder scan PAG parcellations. Because the computation of correlation coefficients between nominally labeled clusters (i.e., clusters 1, 2, 3, 4) in PAG is not trivial, we exhaustively permuted the cluster labels and computed the correlation coefficient for each permutation. Then, we used the highest correlation as a measure of similarity between the empty and full bladder state parcellation. We performed a Fisher transformation to normalize the correlation values to Z-scores.

In order to obtain an indication of how dissimilar patients were from controls, we estimated the distance of each participant (expressed in SEM) to the mean of the rest of the group. Then, we used permutation testing to assess whether this dissimilarity between the participant and the group mean was higher than could be assumed based on chance. We randomly shuffled the group labels (patient or control) 1000 times, and for each permutation we computed the dissimilarity between the permuted patient label and control group, building a distribution under the null hypothesis. We then calculated the *p*-value with the differences in the mean between the two groups. The statistical significance level was set at *p* ≤ 0.05 after correcting for multiple comparisons.

## 3. Results

### 3.1. Participants 

All participants included in the analyses (eight participants; six healthy adults, and two OAB patients) were able to complete the scanning protocol. No adverse events were reported, neither during nor after the MRI experiment. The infused volume for patient 1 (P1) and patient 2 (P2) was 171 mL and 131 mL, respectively, with a voided volume after the scanning procedure of 550 mL (P1), and 275 mL (P2). None of the participants showed any sign of non-voiding contractions or detrusor overactivity on the urodynamic recordings. For each participant, the voided volume after the execution of the scanning protocol was higher than the average volume of voids with an urgency level of 2 reported in the micturition diary. This is an indication that the interoceptive nature of the experiment did not significantly influence the total infused volume during the bladder filling protocol. Suggesting a consistency of the full bladder state between daily life situations and the fMRI study.

### 3.2. Parcellation Analysis

**Figure 1:**
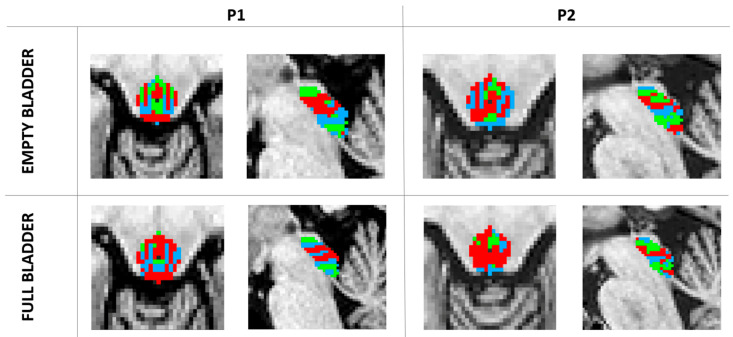
Volume maps. The three PAG clusters were obtained after the Louvain module detection algorithm. Top row: transversal and sagittal view of parcellations from empty bladder state for OAB patient 1 (**P1**) and OAB patient 2 (**P2**). Bottom row: transversal and sagittal view of parcellations from a strong desire to void (full) bladder state. The colors represent corresponding clusters.

Applying the connectivity-based parcellation method (Louvain algorithm) to the fMRI datasets of the participants provided us with a clustering of the PAG for both bladder states for each participant. In both states, we derived three clusters of PAG parcellations for each subject, showing consistency across OAB patients and controls in the parcellation maps of our ROI. The module maps of P1 and P2 can be visualized in Figure 1.

Since we aimed to investigate whether the functional connectivity of the PAG in different bladder states was conserved in OAB patients, we computed the correlation coefficients of the clusters derived from the Louvain algorithm, between the empty and full bladder, to see how similar these subdivisions were. Previous research from our team showed that rs-fMRI parcellations of the PAG, conducted in native space in healthy females, are stable at the within-subject level despite changes in bladder sensations and bladder fullness. [13] In the current study, we obtained high correlation coefficients of PAG parcellations between full and empty bladder in patients with OAB syndrome. The same results were obtained for healthy adults. Since the labeling of the clusters follows a random process, we computed the correlation coefficients for all possible labeling combinations of PAG clusters from each bladder state. The highest correlations for each participant derived from the analysis ranged from 0.8415 to 0.8946. These results support that there is a high agreement in PAG clustering despite different bladder sensations, in both healthy and OAB patients.

### 3.3. Comparison between Patients and Controls

Each patient’s highest correlation value deviated at least 2.2 SEM (P1: −6.0529, P2: 4.9023) from the nearest control (subject 4: 2.6782, subject 3: −1.7622), respectively. Both patients were more extreme than what could be expected based on chance (*p* = 0.0390). Indicating that consistency between empty and full bladder parcellations in OAB patients deviated significantly from controls.

**Figure 2:**
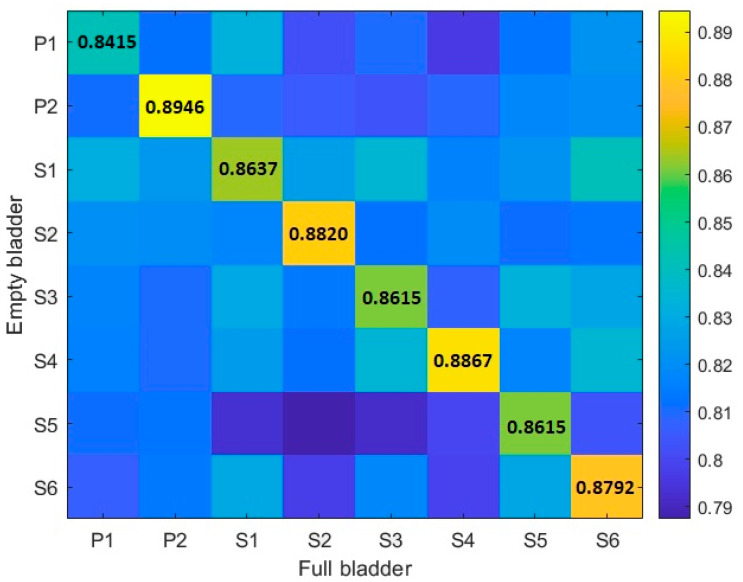
Correlation coefficients. Graphical representations of the matrix obtained of the correlation coefficients of the comparison of PAG resting state functional connectivity in an empty and full bladder. The results of interest are the diagonal of the matrix, which represents the comparison of each state within-subject. P (OAB patient), S (healthy subject).

Although both groups showed high correlations in empty and full bladder states, the coefficients of OAB patients were outliers compared to controls. P1 showed a lower correlation compared to 1000 permutation based on the data from subject 1 to subject 6, while P2 presented a higher correlation compared to subject 1 to subject 6 (Figure 2). 

## 4. Discussion

The current study explored the use of a 7-T fMRI experiment to reliably apply a PAG clustering algorithm in healthy participants and OAB patients during empty and full bladder states. We assessed whether the similarity between clustering outcomes in both states differs between patients and healthy participants. Previous use of this methodological approach was applied effectively to healthy subjects [13,14]. To our knowledge, this is the first time the method is used to parcellate the PAG in OAB patients. Applying this approach to patients allowed us to compare the resting state functional connectivity of this region between healthy and pathological conditions. 

Since the PAG is located in deep regions of the brain and has a small size within the brainstem, ultra-high-field MRI (magnetic field strength ≥ 7-T) is an effective approach to have good spatial resolution and non-invasive research designs to study PAG structure and function.

In agreement with previous investigations, the Louvain module detection algorithm generated three clusters of the PAG for each participant and bladder state and found high similarity between empty and full bladder states. These results suggest that changes in bladder sensations do not influence the resting state functional connectivity in this region and are in line with previous research performed only in healthy females [13]. Parcellations obtained from rs-fMRI experiments are proposed to reflect an underlying anatomical construct [17], and the observed similarity between empty and full bladder parcellations provides support for this notion since the resulting parcellation maps are not significantly affected by bladder volume and sensory state. In contrast with previous studies where PAG masks were personalized [13], the current study not only normalized the data into MNI space but applied a PAG mask generated in MNI space as well. 

To perform the comparison between both groups, the method selected was permutation testing because it only required the assumption of exchangeability [21]. Especially considering the computational power that is currently available, permutation is desired over other conventional tests (i.e., a t-test or f-test). Ludbrook and Dudley discussed that the best procedures for biomedical research with small group sizes are those based on permutation [22].

We showed that the correlations between empty and full bladder parcellations obtained from patients deviated from controls. Although the sample size is small, the results obtained could indicate a trend in differences in resting state functional connectivity between empty and full bladder states when comparing the pathological and healthy state. Therefore, these results are in line with our hypothesis that the consistency of PAG resting state functional connectivity related to bladder sensations (empty and full) in OAB patients is different compared to healthy adults. However, the results were surprising since P1 had a lower correlation compared to controls, and P2 had a higher correlation. One might postulate, that these differences potentially reflect underlying differences in the pathophysiology of the included patients, but this remains for future research to further explore.

Numerous studies have shown abnormalities in brain activity between OAB patients and healthy adults [4,18,23,24,25]. For example, Griffiths et al. suggested that cortical lesions cause bladder control problems [23], while Komesu et al. showed an association between urinary urgency and increased activation of the limbic cortex [24]. However, to our knowledge, no other studies have localized differences in activity to PAG subregions. The results reported here show that our novel methodological approach is a promising method to look at differences in PAG activity. 

Surprisingly enough, Tadic et al. found differences in brain activity between phenotypes of OAB patients. They stated that functional differences observed in their cohort suggest that the OAB syndrome is a heterogeneous clinical condition with a spectrum of functional severity and/or different phenotypes of patients [25].

Despite obtaining promising results, there are several limitations that still prevail. Due to technical difficulties, we were only able to include two OAB patients in the analyses. Further research should include larger cohorts to validate the results found in the study at hand.

Furthermore, future research could implement an additional use of diagnostic tools, such as urodynamics, to understand the underlying pathophysiology of every patient. As suggested by Tadic et al., patients with detrusor overactivity (DO) and more severe functional impairment, as seen in older patients, have a different central nervous system activity than those without DO [25]. Implementing the fMRI approach together with urodynamics will enable better characterization of patients included in the study. These differences in patients could be related to different pathophysiological mechanisms and explain why some patients respond to certain treatments while others do not. If new studies include bigger cohorts, it will be interesting to stratify patients according to different OAB complaints. In-depth evaluation of (sensation-related) micturition diaries may offer a useful tool to further classify patients to better interpret MRI changes. This will not only allow the assessment of bladder capacity, frequency, and natural voiding but also provide additional information on patient characteristics. Ultimately, not only OAB patients could be the subjects for this study method, but any patients with other LUT conditions. 

If specific abnormalities in subregions of PAG regarding functional connectivity patterns are elucidated, this could highlight the problem of OAB patients and use those investigations for the development of novel targets for diagnostic detection or treatment effect follow-up, and lead to more precise therapies. Overall, more research needs to be performed to create better patient selection algorithms and move towards more personalized medicine.

## 5. Conclusions

The present study aimed to assess the within-subject consistency of PAG parcellations between empty and full bladder states and determine whether resting-state functional connectivity of the PAG differs between overactive bladder patients and healthy adults. The results demonstrated that there is a high agreement between parcellations in empty and full bladder states for OAB patients and healthy controls. However, when comparing the consistency of empty and full bladder parcellation maps between patients and controls, even with this small patient number, there were significant differences between groups. 

Ultra-high field fMRI in combination with the novel methodical approach presented in the current study is a promising method to assess the functional organization of the PAG and may, yet, help to understand the underlying pathology and the role of this region in OAB.

## Data Availability

The raw data and custom-made scripts supporting the conclusions of this article will be made available, without undue reservation, upon request to the corresponding author.
